# Supplementary figures and images for: Acupuncture for Depression: A Systematic Review and Meta-Analysis
Source: J Clin Med. 2019 Jul 31;8(8):1140. doi: 10.3390/jcm8081140 (PMC6722678; doi:10.3390/jcm8081140)

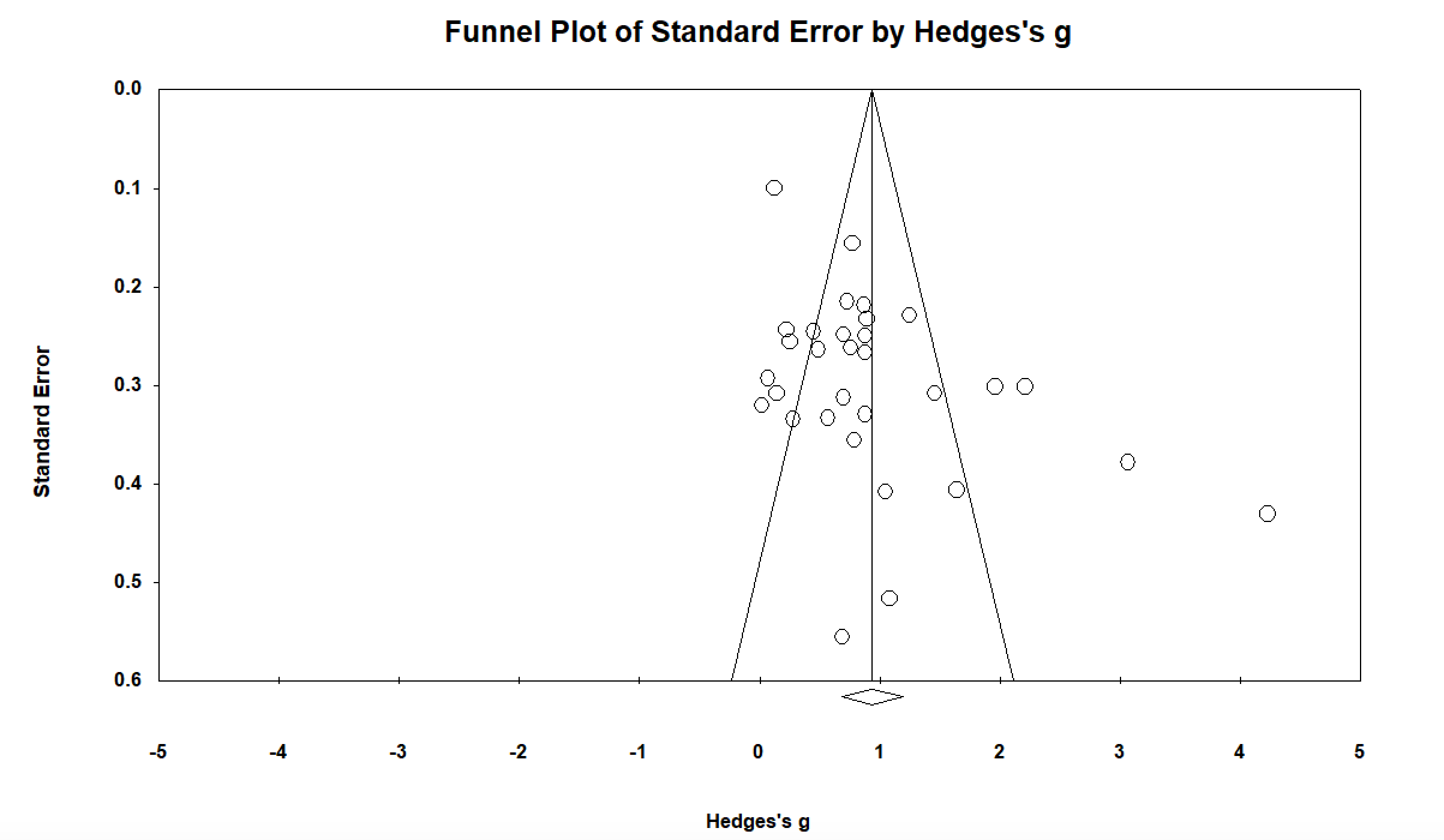

Supplement: Supplementary file 1 [file jcm-08-01140-s001.png]
